# Supplementary material for: Analysis of protein phosphorylation in nerve terminal reveals extensive changes in active zone proteins upon exocytosis
Source: eLife. 2016 Apr 26;5:e14530. doi: 10.7554/eLife.14530 (PMC4894758; doi:10.7554/eLife.14530)
Supplement: Figure 4—source data 1. — DOI: http://dx.doi.org/10.7554/eLife.14530.012 [file elife-14530-fig4-data1.docx]

| **Protein name** | **Gene name** | **Uniprot accession ID** | **Abbreviation** |
| --- | --- | --- | --- |
| Amphiphysin | Amph | F1LPP0 | AMPH |
| Clathrin coat assembly protein AP180 | Snap91 | Q05140-2 | AP180 |
| Dynamin-1 | Dnm1 Dnm | P21575-5 | Dyn1 |
| Dynamin-3 | Dnm3 Dyn3 | Q08877-7 | Dyn3 |
| ELKS/Rab6-interacting/CAST family member 1 | Erc1 | F1LPE9 | ERC1 |
| Epsin-1 (EPS-15-interacting protein 1) | Epn1 | O88339 | Eps1 |
| ERC protein 2 | Erc2 | Z4YNN0 | ERC2 |
| FXYD domain-containing ion transport regulator 7 | Fxyd7 | P59649 | Fxyd7 |
| Intersectin-1 | Itsn1 | D3ZV52 | ITSN1 |
| Liprin-alpha-3 | Ppfia3 | F1LSE6 | Liprin-α3 |
| Plasma membrane calcium-transporting ATPase 4 | Atp2b4 | Q64542-3 | Atp2b4 |
| Protein bassoon | Bsn | G3V984 | Bsn |
| Protein kinase C and casein kinase substrate in neurons protein 1 | Pacsin1 | Q9Z0W5 | PACSIN1 |
| Protein piccolo (Aczonin) | Pclo | Q9JKS6-2 | Pclo |
| Regulating synaptic membrane exocytosis protein 1 | Rims1 | F1LYS1 | RIM1 |
| Regulating synaptic membrane exocytosis protein 2 | Rims2 | D4ABU5 | RIM2 |
| Sodium/potassium/calcium exchanger 2 | Slc24a2 | A0A0A0MXV6 | Slc24a2 |
| Sodium-dependent neutral amino acid transporter SLC6A17 | Slc6a17 Ntt4 Rxt1 | P31662 | Slc6a17 |
| Sodium-driven chloride bicarbonate exchanger | Slc4a10 Ncbe | Q80ZA5 | Slc4a10 |
| Synapsin-1 | Syn1 | P09951 | Syn1 |
| Synapsin-2 | Syn2 | G3V733 | Syn2 |
| Synapsin-3 | Syn3 | A0A096MIT7 | Syn3 |
| Synaptojanin-1 | Synj1 | Q62910-2 | Synj1 |
| Syntaxin-6 | Stx6 | Q63635 | Stx6 |
| Syntaxin-binding protein 5 (Tomosyn-1) | Stxbp5 Llgl3 | Q9WU70-2 | Stxbp5 |
| Voltage-dependent P/Q-type calcium channel subunit alpha-1A | Cacna1a | D3ZRQ6 | Cacna1a |
