# Supplementary material for: Analysis of protein phosphorylation in nerve terminal reveals extensive changes in active zone proteins upon exocytosis
Source: eLife. 2016 Apr 26;5:e14530. doi: 10.7554/eLife.14530 (PMC4894758; doi:10.7554/eLife.14530)
Supplement: Figure 6—source data 1. — DOI: http://dx.doi.org/10.7554/eLife.14530.015 [file elife-14530-fig6-data1.docx]

| **Sequence** | **Name** | **Protein ID** | **Length (aa)** |
| --- | --- | --- | --- |
| SLQRQLSIALPQE | Liprin-a3_S75 | F1LSE6 | 13 |
| SLQRQLAIALPQE | Liprin-a3_S75 | F1LSE6 | 13 |
| MMQRSMSDPKPLS | Pclo_S3626 | Q9JKS6-2 | 13 |
| MMQRSMADPKPLS | Pclo_A3626 | Q9JKS6-2 | 13 |
| TLQRSLSDPKPLS | Bsn_S2844 | G3V984 | 13 |
| TLQRSLADPKPLS | Bsn_A2844 | G3V984 | 13 |
| VPVRSGSIEQASL | Rim1_S1141 | F1LYS1 | 13 |
| VPVRSGAIEQASL | Rim1_A1141 | F1LYS1 | 13 |
| RHERRHSDVALPH | Rim1_S413 | F1LYS1 | 13 |
| RHERRHADVALPH | Rim1_S413 | F1LYS1 | 13 |
| GPIRQASQAGPGP | Syn1_S603 | P09951 | 13 |
